# The relative effectiveness of fiscal and monetary policies in promoting Egypt’s output growth: an empirical investigation using an ARDL approach

**DOI:** 10.1186/s40008-023-00298-8

**Published:** 2023-05-11

**Authors:** Israa A. El Husseiny

**Affiliations:** Economics Department, Faculty of Economics and Political Science, Cairo University, Giza, 12613 Egypt

**Keywords:** Fiscal policy, Monetary policy, Economic growth, ARDL, Egypt, E52, E62, C50

## Abstract

The relative effectiveness of fiscal and monetary policies in promoting economic growth is not sufficiently examined at the empirical level for developing countries, including Egypt in particular. Hence, this paper is the first attempt to empirically examine the relative effectiveness of fiscal and monetary policies in promoting Egypt’s output growth utilizing a time-series data set over the time-period (1960–2019). The study employs the Autoregressive Distributed Lag (ARDL) Bounds testing approach to cointegration to investigate the long run and short run effects of fiscal and monetary policies on Egypt’s output growth under a modified version of the St. Louis equation model. The study finds that both monetary and fiscal policies have a positive impact on the economic activity in the long run. However, while monetary policy seems to be more effective than fiscal policy in stimulating the growth rate of nominal GDP, fiscal policy tends to have a larger, more predictable and faster impact than monetary policy on the real economic activity. Accordingly, Egypt’s policymakers are advised to follow the Keynesian’s prescription in terms of increasing the reliance on fiscal policy compared to monetary policy to achieve macroeconomic stability in both the short run and long run.

## Introduction

Achieving high, stable and sustainable output growth is one of the fundamental goals of any macroeconomic-stabilization policy. Monetary and fiscal policy actions are the two main policy options that governments use to accomplish this policy objective through reacting to the economic conditions (Rahman 2005; Şen and Kaya 2015; Abu Hasan et al. 2016; Usman and ul-Haq 2016; Richard et al. 2018). While monetary policy, managed by the central bank, is mainly concerned with the control of money supply and the management of interest rate and exchange rate, fiscal policy is concerned with how government influences economic output through its expenditure and taxation policy (Mishkin 2012; Usman and ul-Haq 2016; Ajayi and Aluko 2017). The appropriate implementation of the two policies is necessary for achieving macroeconomic stability and strengthening the economic performance (Ajayi and Aluko 2017).

An extensive research has been dedicated to explaining the role of monetary and fiscal policies in stimulating economic growth and achieving macroeconomic stability. Yet, the relative effectiveness of the two policies has always been one of the controversial issues in the contemporary macroeconomic policy debate among academic economists and policy makers at both the theoretical and empirical fronts.

This debate goes back to the 1960s and it constitutes the cornerstone of the ideological difference between the Keynesians and Monetarist economists. On the one hand, the Monetarists believe in the effective role of monetary policy actions in stimulating the economic activity. Hence, they call for abrupt increases of money supply by central banks to boost the output level. On the other hand, the Keynesians strongly support the idea that fiscal policy is more powerful than monetary policy in stimulating the economic activity. The Keynesians base their view mainly on the concept of “liquidity trap” according to which increases in money supply fail to accelerate output and growth, when real interest rate reaches its minimum level. In this case, governments should rely on fiscal policy to stimulate private investment, support aggregate demand, and restore full employment (Abu Hasan et al. 2016).

According to Mundell (1962, p. 70), in a disequilibrium situation “monetary policy ought to be aimed at external objectives and fiscal policy at internal objectives” as doing the opposite would worsen the disequilibrium situation.

The Keynesians-Monetarists debate on the relative effectiveness of monetary and fiscal policies has been articulated in a discussion known as Ando-Modigliani and Friedman-Meiselman (AM-FM) debate, referring to Ando and Modigliani (1965) and Friedman and Meiselman (1963 and 1965). Using the US annual data, Friedman and Meiselman (1963) concluded that money stock matters more than autonomous expenditures for consumption expenditure. In contrary, Ando and Modigliani (1965) contended that the Keynesian “income-expenditure” theory outperforms the “quantity theory of money”.

Later on, an extensive volume of empirical research has been devoted to examine the Keynesians-Monetarists debate under both large-scale structural models and small-scale reduced-form models (Hasan 2001). The empirical findings of both approaches, however, have been mixed and far from confirming on the superiority of either of the two macroeconomic policies.

More specifically, large-scale structural models, such as the Federal Reserve Bank-Massachusetts Institute of Technology (FRB-MIT) model, provide empirical evidence in favor of the Keynesians view. Findings of these models indicate that fiscal policy exerts a relatively more dominant influence on the aggregate economic activity compared to monetary policy in the USA and developed countries (*see for example* Evans and Klien 1967; De Leeuw and Grämlich 1968; Duesenberry et al. 1969; De Leeuw and Kalchbrenner 1969; Modigliani and Ando 1976).^1^ On the other hand, single-equation reduced form models, such as those based on the “St. Louis equation” have supported the Monetarists view. Empirical findings of these models suggest that monetary actions have a larger, more predictable, and faster impact on economic activity than fiscal actions (*see for instance* Andersen and Jordan 1968; Andersen and Carlson 1970; Carlson 1978; Hafer 1982). Proponents of these models suggest that monetary policy is more effective as a driving force behind nominal income, arguing against the effectiveness of fiscal policy due to its inflationary and crowding-out effects (Hasan 2001; Fatima and Iqbal 2003; Rahman 2005; Ali et al. 2008; Richard et al. 2018).

The differences in the empirical results of the relevant studies suggest that the relative effectiveness of monetary and fiscal policies largely depends on the prevailing economic and political conditions and structures, which differ between developed and developing countries as well as within each of these two groups of countries (Richard et al. 2018).

In Egypt, fiscal policy is conducted and managed by the Ministry of Finance (MoF) and it is governed by various relevant Constitutional and legislative articles (i.e., State’s General Budget Law No. 53 of 1973 which has been recently replaced by the Unified Public Finance Law No, 6 of 2022). In general, fiscal policy in Egypt is managed following a “discretionary” rather than a “rule-based” approach. Several procedural rules that govern the roles and responsibilities of the key players as well as the timeline of the different stages of the budget cycle exist. Yet, neither fiscal deficit targets nor sectoral expenditure ceilings have been introduced in Egypt so far. As a result, Egypt’s fiscal performance has been characterized by excessive budget deficits that led to relatively high and increasing levels of public debt (El Husseiny 2023). Additionally, there is lack of empirical evidence that supports the countercyclical behavior of fiscal policy in Egypt (El Husseiny 2018).

At another front, according to Law no. 194 of 2020 of the Central Bank and Banking Sector, the Central Bank of Egypt (CBE) is entrusted with the formulation and implementation of the monetary policy, with price stability being the primary and overriding objective. The CBE is committed to achieving, over the medium term, low rates of inflation needed for maintaining confidence and sustaining high rates of investment and economic growth. Indeed, the CBE intends to put in place a formal inflation targeting framework to anchor monetary policy once the fundamental prerequisites are met.^2^

Recently in 2020, many governments around the globe, including Egypt, have responded to COVID-19 pandemic by introducing sound monetary and fiscal measures and stimulus packages with an aim to support the economic recovery and to mitigate the adverse consequences of the crisis on the livelihoods and the economies.

In particular, Egypt responded to the pandemic by introducing a stimulus package of around 100 billion Egyptian pounds representing almost 2% of GDP. This stimulus package aimed to support the groups and sectors affected by the pandemic, including industry, export, tourism, aviation and contracting. Moreover, the adopted policies were designed to make the economy more resilient in facing similar shocks. The speed with which these policies have been introduced and implemented, along with the effective coordination between the fiscal and monetary measures, have helped mitigate the negative effects of the pandemic. The successful implementation of the macroeconomic stabilization reform program that took place during 2016–2019 has also provided strong and stable economic conditions that allowed for building a relatively diversified and resilient economy. As such, despite the relative economic slowdown during the pandemic period, Egypt remained one of the few countries worldwide and the only country in the Middle East and North Africa region that witnessed positive growth rates during the crisis (i.e., 3.6% in 2019/2020 and 3.3% in 2020/2021) (Ministry of Planning and Economic Development 2021).

In light of this context, this paper aims to examine the relative effectiveness of monetary and fiscal policy actions on output growth in Egypt using the ‘modified’ version of the St. Louis equation model developed by the Federal Reserve Bank of St. Louis of the USA. The study applies the modern techniques of time-series data analysis using data for the time-period 1960–2019. In particular, we employ the Auto Regressive Distributed Lag (ARDL) bounds testing approach to cointegration to examine the dynamic impact of fiscal and monetary policy measures on output growth. To the best of our knowledge, this study is the first attempt to investigate this issue in Egypt in terms of both methods utilized and time-period covered. The paper comprises of five sections including the introduction and the conclusion. Section 2 is allocated to the review of the relevant literature. Section 3 introduces the model specification, data sources, and estimation methods. Section 4 presents and discusses the study’s findings. Finally, Sect. 5 concludes incorporating some relevant policy implications.

## A review of the “monetarists-fiscalists” debate and the relevant empirical studies

The study by Friedman and Meiselman (1963) is one of the main contributions that triggered the researchers’ interest in examining the relative efficacy of monetary and fiscal actions in output growth. In their study, Friedman and Meiselman (1963) predict that the “stock of money” is more critical and statistically significant than “autonomous expenditure” in explaining movements in national income. In contrary, Ando and Modigliani (1965) as well as DePrano and Mayer (1965) argue against the monetarist claim, suggesting that the very high correlation between the stock of money and nominal income or consumption expenditure is to some extent spurious, resulting from misspecifications and/or improper definition of autonomous expenditure. In addition, they highlight that it is incorrect to stress either money sock or autonomous expenditure to the exclusion of the other variable.

The earliest empirical effort to resolve the monetary-fiscal policy debate can be traced back to Andersen and Jordan (1968) who examined the relative importance of monetary and fiscal policy in the economic stabilisation of the United States. In their study, the authors developed and estimated a reduced-form single-equation model referred to as the “Andersen- Jordan (A-J)” equation or the St. Louis equation. This equation estimates, using the Almon lag procedure, the relationship between changes (first difference) in nominal gross national product (GNP) as the dependent variable, and changes in each of the money stock, high-employment government expenditure, and high employment government receipts, as explanatory variables, using quarterly US data for 1952Q1-1968Q2.^3^ According to the findings of their study, Andersen and Jordan (1968) argue that monetary actions have greater, faster and more predictable impact on economic activities, as measured by nominal income, than fiscal actions.

Andersen and Carlson (1970) also used the St. Louis reduced-form single-equation model and find evidence supporting the hypothesis that monetary actions, as measured by changes in the money stock, play a strategic role in stimulating output. In contrary, fiscal actions, as measured by changes in the high-employment Federal expenditures, are found to have insignificant effects especially in the long run.

Gramlich (1971) indicates that while both monetary and fiscal policies matter for the economic activity, money seems to matter greatly. Moreover, the study shows that the significance of the monetary variable tends to decrease while that of the fiscal variables improves when the reduced equation is estimated in real rather than nominal terms, arguing that monetary variables have been benefiting from the fact that the reduced-form equations are usually estimated in current dollars.

Few years later, Benjamin Friedman (1977) estimated an updated version of the St. Louis model using an extended sample of the US data covering the time-period from 1953 to 1976. The study found evidence supporting the statistical significance of fiscal actions for nominal income (GNP). Hence, the author argues that while the relatively strong impact of monetary actions continues to hold, “the St. Louis model now believes in fiscal policy” (p.367). In response to this claim, Carlson (1978) re-estimates the St. Louis equation using the “rate of change” in the variables rather than the “first difference” to avoid the heteroscedasticity problem. The evidence from the updated and corrected estimation, however, does not support Benjamin Friedman’s argument that the St. Louis equation believes in fiscal policy.

Some economic researchers [*see for instance*, Stein 1980; Ahmed and Johannes 1984; Batten and Thornton 1986] have raised criticisms against the use of the St. Louis equation model that make its results biased and inconsistent. The most commonly cited criticisms include the fact that the St. Louis equation is a reduced-form equation and it fails to identify appropriate measures of monetary and fiscal policies making it subject to simultaneous equation bias. Indeed, according to the critiques, the policy variables used in the equation (i.e. money supply and government expenditure) are not statistically exogenous which creates an endogeneity problem. Moreover, it has been argued that the St. Louis equation suffers from the heteroscedasticity problem as well as a specification error since it omits some relevant exogenous variables (e.g., interest rates). Moreover, the constrained Almon lag procedure used in the St. Louis equation has also been under criticism. Furthermore, some argue that the use of the St. Louis equation in developing countries is less relevant since they have low degree of monetization (Rahman 2005; Ajayi and Aluko 2017).

Despite its drawbacks and limitations, the reduced-form single-equation model has formed the basis of a considerable volume of empirical studies that aim to examine the relative effectiveness of monetary and fiscal policy actions in stimulating the economic activity. Yet, the findings of estimating the St. Louis equation-based models, in both developed and developing economies, have been mixed. While a large number of researchers generally find support for the monetarists view, some could find evidence that supports the Keynesians view (Bynoe 1994; Hasan 2001; Abu Hasan et al.; 2016; Ajayi and Aluko 2017).

It is noteworthy that some researchers have introduced several modifications to the St. Louis equation over the years (Ajayi and Aluko 2017). One of the most popular modified versions of the St. Louis equation is the one which was proposed by Darrat (1984) and followed by Chowdhury (1986), as follows:

$${\text{Y}}_{{\text{t}}} \,{ = }\,{\text{C}}_{{0}} \,{ + }\,\mathop \sum \limits_{i = 0}^{3} {\text{m}}_{{\text{i}}} {\text{M}}_{{{\text{t}} - {\text{i}}}} \, + \,\mathop \sum \limits_{i = 0}^{4} {\text{f}}_{{\text{i}}} {\text{F}}_{{{\text{t}} - {\text{i}}}} \, + \,\mathop \sum \limits_{i = 0}^{2} \,{\text{e}}_{{\text{i}}} {\text{E}}_{{{\text{t}} - {\text{i}}}} \, + \,{\text{U}}_{{\text{t}}}$$

where growth rate of nominal income “Y” is regressed on growth rate of narrow money supply “M”, growth rate of government expenditure “F”, and growth rate of export receipts “E”. Estimating this equation using annual data of Bangladesh over the time-period 1972–1983, Chowdhury (1986) finds that government expenditure has greater, longer and more predictable impact on nominal income than money stock.

A review of a sample of empirical studies that have estimated different versions of the St. Louis equation model to examine the relative effectiveness of monetary and fiscal policies reveals several issues that worth highlighting. First, in terms of the methods utilized, most of the reviewed studies employ various techniques of time-series data analysis, such as Vector Auto Regression (VAR); Johansen Cointegration test; Auto Regressive Distributed Lag (ARDL) bounds test approach to cointegration, and Error Correction Model (ECM). Out of the reviewed studies, Ali et al. (2008) is the only one to examine the issue using panel data analysis (i.e. panel ARDL).

Second, in terms of the variable used as a measure of economic growth, the majority of reviewed studies uses nominal GDP as the dependent variable (*see for instance*, Orsmond 1992; Bynoe 1994; Fatima and Iqbal 2003; Ali et al. 2008; Mahmood and Sial 2011; Abu Hasan et al. 2016; Ajayi and Aluko 2017; Richard et al. 2018). Some researchers, however, such as Rahman (2005); Hussain (2014); Şen and Kaya (2015); Özer and Karagöl (2018); and Tarawalie and Kargbo (2020), have used real GDP instead. Hasan (2001) examines two specifications of the estimated model, where both nominal GDP and real GDP are used, respectively, to proxy economic growth.

Third, regarding the variables used as measures of monetary and fiscal policy actions, the majority of reviewed studies uses money supply, narrowly or broadly defined, as proxy of monetary actions and government expenditure as proxy of fiscal actions. Yet, in some studies, variables like government revenues and/or fiscal deficit are used in combination with or in replacement of government expenditure to proxy fiscal policy actions (s*ee for instance*, Ali et al. 2008; Iyeli et al. 2012; Şen and Kaya 2015; Abu Hasan et al. 2016; Usman and ul-Haq 2016; Richard et al. 2018; Tarawalie and Kargbo 2020). Similarly, some studies add various measures of monetary policy, such as interest rate, exchange rate, inflation, and foreign reserves, in combination with or in replacement of money supply to proxy monetary actions (*see for instance*, Rahman 2005; Hussain 2014; Şen and Kaya 2015; Usman and ul-Haq 2016; Richard et al. 2018; Özer and Karagöl, 2018; Tarawalie and Kargbo 2020). Beside the basic fiscal and monetary policy measures, other control variables are included in some of the reviewed studies, the most important of which is export receipts, which is used to proxy the autonomous expenditure on international trade.

Fourth, when it comes to how variables in the estimated equation are measured, two main approaches dominate the literature. Namely, the variables could be expressed in their “level” form (*e.g.* Hasan 2001; Hussain 2014; Abu Hasan et al. 2016; Özer and Karagöl, 2018; Richard et al. 2018; Tarawalie and Kargbo 2020), or in their “first difference” or “rate of change” form (e.g. Orsmond 1992; Bynoe 1994; Rahman 2005; Ajayi and Aluko 2017). In addition, some studies measure the fiscal and monetary variables expressed as percentage of GDP, such as Mahmood and Sial (2011); Şen and Kaya (2015); and Usman and ul-Haq (2016).

Fifth, the findings of the reviewed studies indicate that the relative impact of monetary and fiscal policies on economic activities differs not only from country to another within a given sample, but also according to whether the impact is examined in the short run or long run.

## Model specification, data sources, and estimation methods

In this section, we introduce the model specification, data sources and estimation methods utilized to investigate the relative effectiveness of monetary and fiscal policy actions in promoting output growth in Egypt during the time-period spanning from 1960 to 2019.

### Model specification and data sources

To empirically examine the relative effectiveness of fiscal and monetary actions for Egypt’s output growth, we follow the specification utilized by Darrat (1984) and Chowdhury (1986) which introduces two main modifications to the original St. Louis equation model that appeared in Andersen and Jordan (1968). Firstly, all variables are measured in “growth rate” form rather than “first difference” form. As noted by Carlson (1978), this practice is ought to solve the potential heteroscedasticity problem that might exist in the original version of the St. Louis equation. Secondly, the “exports” variable is added to consider the autonomous expenditure component of international trade.

As such, our multivariate regression model is specified as follows:

1 $$ngdp\_g \, = \, f \, \left( {ngov\_cons\_g; \, bm\_g; \, nexport\_g} \right)$$

where *“ngdp_g”* is the growth rate of nominal GDP used as a measure of economic growth. The two variables “*ngov_cons_g”* and “*bm_g*” refer to the growth rate of nominal government consumption expenditure and the growth rate of nominal broad money supply, used as proxies for fiscal and monetary actions respectively. Additionally, “*nexport_g”* refers to the growth rate of export receipts in nominal terms. It is noteworthy that the specification we follow in this study is adopted, albeit with slight modifications, by Bynoe (1994), Fatima and Iqbal (2003); and Ali et al. (2008).

The “exports” variable is used in our analysis to represent the foreign trade sector as it acts as a proxy for the autonomous expenditure component of international trade. According to Darrat (1984), the original specification of the St. Louis equation model implicitly assumes that the economy under consideration is closed. This makes the original specification inappropriate for developing countries, whose economies are largely influenced by the foreign sector. Hence, Darrat (1984) suggested including “exports” as an additional explanatory variable in the relevant models, especially those estimated for developing countries.

Accordingly, several empirical studies on developing countries have considered the “exports” variable beside the fiscal and monetary variables in the relevant analyses to highlight the role of export demand as a driving force of economic growth. Examples of these studies include Darrat (1984), Chowdhury (1986), and Hasan (2001) on Bangladesh; Bynoe (1994) on a set of five African countries; Fatima and Iqbal (2003) on a set of five Asian developing countries; and Ajayi and Aluko (2017) on Nigeria. Indeed, the latter study provides empirical evidence against the claim that “exports” is redundant in the application of the St. Louis equation to the Nigerian economy. Furthermore, empirical studies show that “exports” is among the variables that encounter a significant impact on Egypt’s economic growth in both the short and long run, supporting the “export-led growth hypothesis” (see for instance Khashaba 2010; Torayeh 2011).

In addition to the above-mentioned specification, we run another specification of our model where all variables enter in their natural logarithmic (real-level) form, as follows:

2 $$ln\_rgdp \, = \, f \, \left( {ln\_rgov\_cons; \, ln\_rbm; \, ln\_rexport} \right)$$

where “*ln_rgdp*” is the natural logarithm of real GDP; “*ln_rgov_cons*” is the natural logarithm of real government final consumption expenditure; “*ln_rbm*” is the natural logarithm of real broad money supply; and “*ln_rexport*” is the natural logarithm of real export receipts.

It is noteworthy that Hussain (2014), Özer and Karagöl (2018), and Tarawalie and Kargbo (2020) have estimated a similar specification.

The main purpose of running two specifications of our model is to test the hypothesis of Gramlich (1971) that the relative importance of fiscal and monetary actions for GDP growth differs based on whether the variables are measured in nominal or real terms.

Data on the mentioned variables is extracted from the World Bank’s World Development Indicators (WDI) database for Egypt over the time-period (1960–2019). Table 1 summarizes the study’s variables describing how they are measured. In addition, Table 2 in the Appendix presents the descriptive statistics of the examined variables.

**Table 1:** Measurement and data sources of the study’s variables

Variable symbol	Description/measurement	Data source
*Specification 1*		
* ngdp_g*	Annual growth rate of GDP in current prices	Calculated by the author based on the World Bank’s data on GDP in current LCU
* ngov_cons_g*	Annual growth rate of general government final consumption expenditure in current prices	Calculated by the author based on the World Bank’s data on general government final consumption expenditure in current LCU
* bm_g*	Annual growth rate of broad money	The World Bank
* nexport_g*	Annual growth rate of exports of goods and services in current prices	Calculated by the author based on the World Bank’s data on exports of goods and services in current LCU
*Specification 2*		
* ln_rgdp*	Natural logarithm of GDP in constant prices	Calculated by the author based on the World Bank’s data on GDP in constant LCU
* ln_rgov_cons*	Natural logarithm of general government final consumption expenditure in constant prices	Calculated by the author based on the World Bank’s data on general government final consumption expenditure in constant LCU
* ln_rbm*	Natural logarithm of broad money in constant prices	Calculated by the author based on the World Bank’s data on broad money in current LCU and the Consumer Price Index (CPI)
* ln_rexport*	Natural logarithm of exports of goods and services in constant prices	Calculated by the author based on the World Bank’s data on exports of goods and services in constant LCU

### Estimation methods

The current study applies the Autoregressive Distributed Lag (ARDL) Bounds testing approach to examine the existence of a long run cointegration equilibrium relationship between the study’s variables. As developed by Pesaran and Shin (1999) and later expanded by Pesaran et al. (2001), the ARDL approach has multiple advantages over the other alternative methods of testing cointegration, including Johansen cointegration test. On top of these advantages is that the ARDL allows for using variables of different integration orders in analysis (i.e. a mix of stationary and non-stationary variables). In addition, this approach allows for estimating the short run and long run forms simultaneously by forming an Error Correction Model (ECM) derived from the ARDL model (Özer and Karagöl, 2018). Furthermore, the ARDL approach provides unbiased estimates of the long run model even when some of the regressors are endogenous. Unlike most of the conventional cointegration procedures which produce valid estimates only for large sample size, this approach is believed to be suitable for small sample size as well (Udoh et al. 2015).

Before proceeding with the estimation of the ARDL model, we first test the stationarity of the variables’ time-series at their levels, using the Dickey-Fuller (DF), Augmented Dickey-Fuller (ADF) and Phillips-Perron (PP) tests.

According to these tests, a given variable is considered to be stationary or I(0) if the test’s null hypothesis is rejected, indicating to the absence of a unit root in the variable. In contrary, the variable is considered non-stationary or integrated of an order greater than zero if we fail to reject the null hypothesis, which indicates to existence of a unit root. The integration order of non-stationary variables can then be determined by running the test at the first-difference form of those variables. After identifying the order of integration for all variables covered by analysis, the ARDL F-bounds test can be run.

Based on Eq. (1), the long run relationship between annual growth rate of nominal GDP “*ngdp_g”*; annual growth rate of nominal general government final consumption expenditure* “ngov_cons_g”*; annual growth rate of broad money stock *“bm_g”*; *and* annual growth rate of nominal export receipts* “nexport_g*” can be represented by the following linear form:

3 $$ngdp\_g_{t} = \beta_{0} + \beta_{1} ngov\_cons\_g_{t} + \, \beta_{2} bm\_g_{t} + \beta_{3} {nexport}\_g_{t} + \varepsilon_{t}$$

where *t* refers to the time period (i.e. year); *ε* represents the error term, *β*_*0*_ is the constant term*,* and *β*_*i*_* (where i* = *1, 2, 3)* represent the coefficients of the examined explanatory variables.

The unrestricted error correction representation of the ARDL framework of Eq. (3) can be given by Eq. (4) as follows:4where Δ represents the first difference operator; δ_1_, δ_2_, δ_3_, δ_4_, are the long run parameters; and  is the optimal lag minus 1.

According to the ARDL approach, testing for the existence of a long run cointegration relationship between the explanatory variables and the dependent variable requires as a first step estimating Eq. (4) by Ordinary Least Squares (OLS). In a second step, an F-test or Wald test is carried out for the joint significance of the coefficients of the lagged levels of the variables. In particular, the null hypothesis of non-existence of cointegration among the variables in Eq. (4) (H_0_: *δ*_*1*_ = *δ*_*2*_ = *δ*_*3*_ = *δ*_*4*_ = 0) is tested against the alternative hypothesis of having cointegration (H_1_: *δ*_*1*_* ≠ δ*_*2*_* ≠ δ*_*3*_* ≠ δ*_*4*_* ≠ *0). As such, a rejection of the null hypothesis indicates to the existence of a long run cointegration relationship between the examined variables.

The outcome of the test is determined by comparing the calculated F-statistic value of the Wald test with two sets of tabulated critical bound values established by Pesaran et al. (2001). The lower critical bound assumes that all the variables are I(0) while the upper bound assumes that all the variables are I(1). If the F-statistic value exceeds the upper bound critical value at any given level of significance, the null hypothesis of non-existence of cointegration will be rejected at that level of significance. If, instead, the calculated F-statistic value is found to be less than the lower bound critical values, the null hypothesis of absence of a cointegration between the variables cannot be rejected. Finally, if the calculated F-statistic value falls between the lower and upper critical bound values, the test result will be inconclusive. In this case, the statistical significance of the coefficient of the speed of adjustment may give an indication on the existence of a cointegration relationship between the examined variables (Özer and Karagöl 2018).

If a long run relationship between the variables is established, a short run (error correction) version of the ARDL model can be represented by Eq. (5) as follows:5where *ecm*_*t*−1_ is the error correction model term which reflects the speed of adjustment to reach the long run equilibrium following a short run shock. As such, the coefficient of this term has to be negative and statistically significant.

For the diagnostic checking, we test for the normal distribution of residuals as well as for the absence of serial correlation and heteroscedasticity in the residuals. In addition, we check the stability of the model by using Ramsey RESET test as well as the two tests of Cumulative Sum of Recursive Residuals (CUSUM) and Cumulative Sum of Square of Recursive Residuals (CUSUMSQ).

We repeat the whole preceding analysis for the second specification of our model that is represented by Eq. (2).

## Findings and discussion

In this section, we present and discuss the findings of our empirical analysis undertaken to assess the relative effectiveness of fiscal and monetary policies in promoting Egypt’s output growth.

### Unit root tests findings

Table 3 in the Appendix presents the results of DF, ADF and PP Unit Root tests. We can conclude from the results that the explanatory variables that reflect the fiscal action and the exports, in the first specification, namely “*ngov_cons_g”* and “*nexport_g”,* are stationary at levels, that is I(0), at 1% level of significance. Yet, the variable that measures the monetary action “*bm_g”* is found to be non-stationary at level, but stationary at the first difference, that is I(1), at the 1% level of significance. While the dependent variable under specification 1, “*ngdp_g”,* is found to be I(1) using the DF test, it is found to be I(0) according to the results of the ADF and PP tests.

Considering the variables used in the second specification, the results indicate that all of them are non-stationary at levels but are stationary at the first difference, at 1% level of significance, suggesting that they are all integrated of order 1.

Since the variables of the first specification have different degrees of integration and those of the second specification are all integrated of the same order, the decision of applying the ARDL Bounds test approach to cointegration, to both specifications, could be justified.

### ARDL findings

In the following sub-sections, we present the findings of the estimated ARDL model under its two specifications.

#### Specification 1: nominal variables

The ARDL Bounds test is carried out for the first specification of our model based on the estimated unrestricted error correction model, represented by Eq. (4), with automatic lag selection. The test is carried out using E-views software, version 11 and the results are shown in Table 4 in the Appendix.

The results reveal that the F-statistic value exceeds the tabulated upper-bound critical value at 1% level of significance. Hence, the test’s null hypothesis can be rejected indicating to the existence of a long run equilibrium (cointegration) relationship among the examined variables. As such, the estimates of the long run and short run parameters, as presented in Table 5 in the Appendix, can be used for further analysis.

The estimation results of the long run relationship indicate that the coefficients of the three explanatory variables included in the model encounter the positive expected sign. Yet, while the coefficients of the two variables that reflect the monetary action and the export receipts are significant at the 1% level of significance, the variable that measures the fiscal action is significant only at the 10% level. This reflects the relatively poor significance of the fiscal action’s impact on the economic activity in the long run.

Following the approach utilized by the previous studies (*see for instance* Andersen and Jordan 1968; Orsmond 1992; Hasan 2001; Tarawalie and Kargbo 2020), the relative effectiveness of monetary and fiscal actions in stimulating economic growth is evaluated based on three criteria, namely the “magnitude”, the “predictability” and the “speed” of the impact of these two policies on economic growth.

Considering the first criterion, “predictability” of the response of economic activity to a given policy action can be measured by the t-statistic value of the coefficient of the variable measuring that action, that is the value of the variable’s coefficient relative to the value of its standard error (Andersen and Jordan 1968). The larger the value of the t-statistic of a given explanatory variable’s coefficient, the greater the predictability of this variable’s impact on the dependent variable. The t-statistic values of the coefficients of the three variables examined in our model are as follows: 4.15 for the monetary action variable, 3.48 for the exports variable, and 1.98 for the fiscal action variable. As such, based on the “predictability” criterion, we can conclude that the response of the economic activity to the monetary action in the log run is more predictable than the response of the economic activity to the fiscal action. In other words, under this specification of our model, the impact of the monetary policy on the economic activity, measured by the growth rate of nominal GDP, is believed to be more predictable than that of the fiscal policy.

As for the “magnitude” of the influence of monetary and fiscal actions on economic activity, an examination of the absolute size of the regression coefficient of the monetary action relative to that of the fiscal action is required. The larger the size of the coefficient of a given policy variable, regardless its sign, the greater the magnitude of its impact on the economic activity. Yet, since variables have different time dimensions and are a mixture of stocks and flows, a common approach is to change the regression coefficients to “beta coefficients”. In this case, the beta coefficient considers the past variation of changes in each explanatory variable relative to the past variation of changes in the dependent variable (i.e.GDP). The size of the beta coefficients can then be directly compared to measure the relative contribution of each explanatory variable to variations in the dependent variable (Andersen and Jordan 1968).

For a given independent variable, the beta coefficient is calculated as the product of the estimated regression coefficient and the ratio of the standard deviation of the independent variable to the standard deviation of the dependent variable (Hasan 2001).

Based on the estimation results of the long run relationship as expressed in Table 5 of the Appendix, we measure the beta coefficients of the three examined explanatory variables.

The beta coefficients are estimated to be 0.52, 0.44, and 0.23 for the money supply, exports, and government expenditure variables, respectively. Accordingly, we can conclude from the results of beta coefficients that the monetary policy variable exerts the dominant impact on nominal GDP growth in the long run followed by the exports variable, whereas the fiscal policy variable has the least effect.

Regarding the “speed” criterion of the relative impact of monetary and fiscal policy actions on economic activity, this can be tested by examining the characteristics of the lag structure in the estimated regression (Andersen and Jordan 1968). Hence, this criterion is relevant to the short run rather than the long run form of the ARDL approach, as the former shows the distributed lags.

The short run (ECM) results of the first specification, as presented in Table 5 in the Appendix, reveal that the monetary policy variable has a significant impact on the change in nominal GDP growth rate in the first lag, yet this impact is negative. Both the fiscal policy variable and the exports variable are dropped from the short run form according to the automatic lag selection procedure. Moreover, the coefficient of the ecm(-1) which measures the speed of adjustment is significant and has the expected negative sign, confirming that there exists a long run causality relationship between the examined variables. The absolute value of this coefficient indicates that around 85% of the disequilibrium from the long run equilibrium path in any given period will be corrected in the following period. Hence, it takes almost 1.18 years to adjust from a shock and reach the long run equilibrium path.

Based on our estimation results of the first specification, we can argue that monetary policy seems to be more effective than fiscal policy in stimulating the economic activity in Egypt.

According to this specification, the impact of the monetary policy action on the growth rate of nominal GDP is larger and more predictable than that of the fiscal policy action.

#### Specification 2: real variables

The results of the ARDL Bounds test of specification 2, in which “real” rather than “nominal” variables are used, are shown in Table 4 in the Appendix. The results reveal that the F-statistic value exceeds the tabulated upper-bound critical value at the 1% level of significance, indicating to the rejection of the test’s null hypothesis. Accordingly, we conclude that there exists a long run equilibrium (cointegration) relationship among the examined variables under this specification as well. Table 6 in the Appendix presents the results of the long run and short run forms of the second specification of our model.

The estimation results of the long run relationship indicate that the coefficients of the three explanatory variables included in the model encounter the positive expected sign and are highly significant, reflecting the importance of the fiscal policy, monetary policy, and exports for the real economic activity in Egypt.

Considering the “predictability” criterion of the relative effectiveness of monetary and fiscal actions on economic growth, the t-statistic values of the coefficients of the three variables included in this specification are 5.6 for the fiscal policy variable, 4.5 for the monetary policy variable, and 4.1 for the exports variable. Hence, we can conclude that under this specification, the response of the real economic activity to fiscal policy is more predictable than its response to the monetary policy in the long run.

Regarding the “magnitude” of the relative influence of the various policy actions on the economic activity, the results of the long run form show that the estimated coefficient of the fiscal policy variable is the largest followed by the one of the monetary policy variable. Based on the estimation results, the calculated beta coefficients of the three examined explanatory variables are as follows: 0.44 for the fiscal policy variable, 0.30 for the monetary policy variable, and 0.28 for the exports variable. As such, we conclude that under this specification, fiscal policy exerts the dominant influence on real GDP in the long run followed by monetary policy, whereas exports has the least effect.

The short run (ECM) results of the second specification, as presented in Table 6 in the Appendix, reveal that the three examined explanatory variables exert a significant impact on the real economic activity in the short run. While the fiscal policy variable has a positive and statistically significant coefficient in lag (0) and lag (1), it has a negative one in lag (2). In contrary, the monetary policy variable has a negative coefficient in lag (1) and a positive one in lag (3). As for the exports variable, the short run results indicate that this variable exerts a positive and significant impact on the real economic activity in lag (0) while it has a negative impact in lags (1&2). Moreover, the coefficient of the ecm(-1) which measures the speed of adjustment is significant and has the expected negative sign. The absolute value of this coefficient indicates that the system is stable and that around 19% of the disequilibrium from the long run relationship in any given period will be corrected in the next period. In other words, it could take almost 5 years to fully adjust from a given shock and reach the long run equilibrium path.

Considering the “predictability” criterion of the relative effectiveness of the policy actions on real economic activity, the short run results indicate that the absolute value of the t-statistic of the fiscal variable’s coefficient is larger than the corresponding values of the monetary and exports variables in each of the comparable time-periods. Hence, we conclude that the impact of the fiscal policy on real economic activity is more predictable than the impact of the monetary policy in the short run.

Regarding the “magnitude” of the relative impact on real GDP of fiscal policy compared to monetary policy, we calculate the beta coefficients for each of the three explanatory variables in each single period based on the values of the estimated significant coefficients in the short run form. Then, we sum up the calculated beta coefficients for each variable to measure the magnitude of its impact on real GDP compared to the other variables. The sum of beta coefficients are as follows: 0.14 for the fiscal policy variable, -0.02 for the monetary policy variable, and -0.05 for the exports variable. As such, the short run impact of fiscal policy on real economic activity is larger compared to the impact of the monetary policy.

Examining the characteristics of the lag structure in the short run form shows that the impact of fiscal policy on real economic activity tends to be “faster” than the impact of monetary policy. A change in the real government final consumption expenditure has a significant impact on the change in real GDP in the same period and in the subsequent two periods. In contrary, a change in the real broad money supply significantly affects the change in real GDP only after one period. In addition, the significant coefficient of the monetary policy variable in the third lag indicates that it takes relatively long time for the monetary action to be reflected in the real economic activity.

**Fig. 1:**
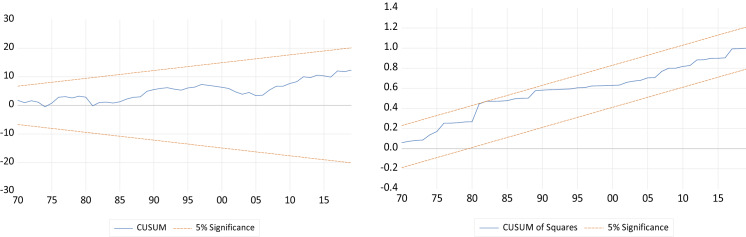
CUSUM and CUSUM of Squares Test Results: Specification 1

**Fig. 2:**
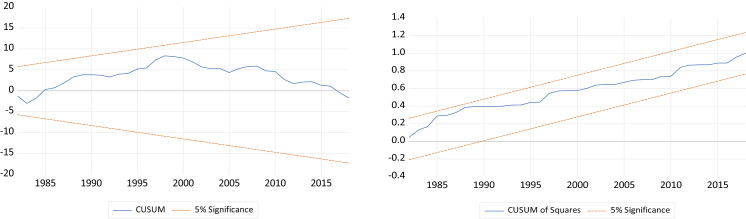
CUSUM and CUSUM of Squares Test Results: Specification 2

The results of the diagnostic tests of the two specifications of our model, as shown in Tables 5 and 6 in the Appendix, respectively, confirm the robustness of the estimates, as they do not indicate any evidence of autocorrelation, heteroscedasticity, or non-normal distribution of residuals. In addition, the null hypothesis of Ramsey RESET test cannot be rejected implying that the model is well specified. Finally, the results of CUSUM and CUSUMSQ tests, as shown by Figs. 1 and 2 in the Appendix, prove the stability of the model’s estimated coefficients, under the two specifications, and that they are within the critical bounds at 5% level of significance.

Based on our estimation results, we can argue that both of monetary and fiscal policies exert a positive and significant impact on the economic activity of Egypt in the long run. Yet, the relative importance of each of the two policies depends on the specification of the estimated model, in terms of how the variables are measured. In particular, when annual growth rate of nominal GDP is used as a measure of the economic activity and all explanatory variables are measured in nominal terms, we find empirical evidence supporting the argument that monetary policy plays the dominant role over fiscal policy in stimulating the economic activity. Hence, the results of this specification prove the validity of the St. Louis equation model in the context of the Egyptian economy. A large number of empirical studies, including Bynoe (1994); Ali et al. (2008); Iyeli et al. (2012); Abu Hasan et al. (2016); Ajayi and Aluko (2017); and Richard et al. (2018) support this finding.

However, when real GDP is used as the dependent variable and all explanatory variables are measured in real terms rather than nominal terms, we find evidence that supports the hypothesis that fiscal policy tends to have a larger, more predictable, and faster impact on the economic activity compared to the monetary policy. Our findings are compatible with those of Hasan (2001) as well as Bölükbaş (2016) and Özer and Karagöl (2018) who found that fiscal policy is stronger than monetary policy in the context of affecting the real economy in Bangladesh and Turkey, respectively. The study of Hussain (2014) has also found that fiscal policy has a more powerful effect on real GDP than monetary policy in Bangladesh, India, and Nepal.

As such, our findings support both the Keynesians and Monetarists views on the relative importance of fiscal and monetary policies for economic activity. While the monetary policy action seems to have the dominant role in stimulating the nominal GDP growth rate in Egypt, the fiscal action is found to play the potent role in stimulating the real economic activity. Hence, our findings definitely support the argument of Gramlich (1971) that the significance of the monetary variable tends to decrease while that of the fiscal variable improves when the reduced equation is estimated in real rather than nominal terms.

## Conclusions

In this paper, we examine the relative effectiveness of monetary and fiscal policies in promoting output growth in Egypt, over the period from 1960 to 2019, using the Auto Regressive Distributed Lag (ARDL) Bounds testing approach to cointegration. In particular, we assess the relative impact of monetary and fiscal policy actions on Egypt’s economic growth in terms of the “magnitude”, “predictability”, and “speed” of that impact, within the framework of the modified St. Louis equation model.

We run our model under two different specifications. In the first specification, we follow a modified version of the St. Louis equation model, where annual growth rate of nominal GDP is regressed on the annual growth rate of each of government final consumption expenditure, broad money supply, and export receipts, measured in nominal terms. In the second specification, we regress the natural logarithm of real GDP on the natural logarithm of the three variables of real government final consumption expenditure, real broad money supply, and real export receipts. The findings of the ARDL Bounds test under the two specifications indicate to the existence of a long run “cointegration” relationship between the examined variables. In addition, the coefficients of the three explanatory variables are found to have the expected positive sign in the long run form, indicating to the positive impact of the monetary and fiscal policy actions as well as export receipts on Egypt’s economic activity. We find, however, that the relative effectiveness of monetary and fiscal policies in promoting output growth depends on the model specification adopted.

Our estimation results based on the first specification support the “Monetarists” view that monetary policy is more effective than fiscal policy in promoting output growth. The impact of the monetary policy on the annual growth rate of nominal GDP is found to be larger and more predictable than the impact of the fiscal policy in both the long run and short run. These findings support a large strand of literature that is based on the St. Louis equation model.

Yet, under the second specification, where real rather than nominal variables are used, we find empirical evidence that supports the “Keynesians” view on the relative importance of fiscal policy over monetary policy in achieving economic stability. In particular, by examining the long run and short run forms of the model under this specification, we find that fiscal policy exerts a larger, more predictable and faster impact on real GDP than monetary policy.

The findings of the empirical work presented in this paper prove that both monetary and fiscal policies have the potential to promote Egypt’s economic activity and achieve macroeconomic stability in the long run. Yet, policy makers are advised to give a higher priority to fiscal policy over monetary policy, if the objective is to promote real economic activity. Increasing “real” government final consumption expenditure tends to have a positive impact on real GDP that is larger, faster, and more predictable than the impact of increasing real broad money supply.

These findings are of a particular importance for the Egyptian economy, especially in the era of the global recession induced by the COVID-19 pandemic. The Government of Egypt has introduced several measures to mitigate the adverse consequences of the crisis, mainly on the poor and the most vulnerable. These measures include both government expenditure increases and tax cuts as well as multiple reductions in the interest rate to boost private investment. In light of this study’s findings, we argue that fiscal policy measures would be relatively more effective than monetary actions in stimulating the real economic activity of Egypt both in the long run and short run. Hence, the Government should rely largely on the fiscal policy, rather than monetary policy, as a powerful toll to enhance macroeconomic stabilization and economic growth. In addition, a proper and sound coordination between the fiscal and monetary policies need to be in place due to their significant impact on Egypt’s economic activity.

The current analysis provided in this paper can be extended in several ways in the future research. First, researchers may replicate the same analysis using quarterly, rather than annual, data of the examined variables to check if data frequency has any effect on the results. Second, researchers may consider the utilization of large-scale structural models in examining a similar research question whether for the Egyptian economy or for any other developing country. Third, different methods of testing for cointegration among variables, other than the ARDL approach, can be used in the analysis.

## Data Availability

The data used in this paper is extracted from the World Development Indicators (WDI) database of the World Bank.

## Appendix

See Tables. 2, 3, 4, 5, 6

**Table 2:** Descriptive statistics of the study’s variables

	*Specification 1*				*Specification 2*			
	*ngdp_g*	*ngov_cons_g*	*bm_g*	*nexport_g*	*ln_rgdp*	*ln_rgov_cons*	*ln_rbm*	*ln_rexport*
Mean	15.14	13.68	16.93	16.81	27.58	25.40	21.84	25.06
Median	14.74	13.85	15.77	10.69	27.71	25.46	22.12	25.12
Maximum	37.09	28.77	51.42	95.83	28.98	26.63	23.42	27.31
Minimum	0.17	− 3.44	1.54	− 29.75	26.01	23.62	19.81	23.21
Standard Deviation	7.44	5.86	9.51	23.72	0.90	0.83	1.11	1.22
No. of observations	59	59	59	59	60	60	59	60

**Table 3:** DF, ADF and PP unit root tests

Null hypothesis: the variable has a unit root							Integration order
Trend specification: trend and intercept for levels and first difference							
Variables	Dickey-fuller (DF)		Augmented dickey-fuller (ADF)		Phillips-perron (PP)		
	Level	First difference	Level	First difference	Level	First difference	
	t-stat	t-stat	t-stat	t-stat	t-stat	t-stat	
Specification 1							
*ngdp_g*	− 2.022	− 9.031 ***	− 4.942***	–	− 5.224***	–	I(1); I(0); I(0)
*ngov_cons_g*	− 7.449***	–	− 7.363***	–	− 7.398***	–	I(0); I(0); I(0)
*bm_g*	− 3.237**	− 9.429 ***	− 3.236*	− 9.723***	− 3.254*	− 9.780***	I(1); I(1); I(1)
*nexport_g*	− 5.985***	–	− 6.105***	–	− 6.076***		I(0); I(0); I(0)
Specification 2							
*ln_rgdp*	− 1.766	− 4.696***	− 1.728	− 4.658***	− 1.287	− 4.667***	I(1); I(1); I(1)
*ln_rgov_cons*	− 0.968	− 10.162***	− 2.906	− 10.214***	− 3.275	− 10.576***	I(1); I(1); I(1)
*ln_rbm*	− 1.485	− 4.573***	− 1.473	− 4.507***	− 1.414	− 4.476***	I(1); I(1); I(1)
*ln_rexport*	− 2.443	− 6.973***	− 2.805	− 7.124***	− 2.932	− 7.121***	I(1); I(1); I(1)

(*), (**), and (***) denote the rejection of the Null Hypothesis, of having a unit root, at 10%, 5%, and 1%, levels of significance, respectively

Order of integration is specified for each variable according to the results of the three stationarity tests at 1% level of significance

**Table 4:** Results of the ARDL Cointegration (F-Bounds) Test

Null Hypothesis: No levels relationship					
Model	Optimal lag length	F-statistic	Bound critical values at 1% level		Outcome
			I (0)	I (1)	
Specification 1					
* ngdp_g* = *f(ngov_cons_g; bm_g; nexport_g)*	(1,0,2,0)	22.211^a^	3.65	4.66	Cointegration exists between the examined variables
Specification 2					
* ln_rgdp* = *f(ln_rgov_cons; ln_rbm; ln_rexport)*	(4,3,4,3)	6.169864^a^	3.65	4.66	Cointegration exists between the examined variables

^a^denotes the rejection of the Null Hypothesis at 1% level of significance

**Table 5:** Long run and short run forms: specification 1

Variable	Coefficient	t-statistic	Prob. Value
Long run results: case 2: restricted constant and no trend			
Dependent variable: *ngdp_g*			
*ngov_cons_g*	0.293688*	1.979902	0.0532
*bm_g*	0.407245***	4.154433	0.0001
*nexport_g*	0.137902***	3.484824	0.0010
*constant*	2.017752	0.805266	0.4245
EC = *ngdp_g-(0.2937*ngov_cons_g* + *0.4072*bm_g* + *0.1379*nexport_g* + *2.0178)*			
Short run results (ECM Form): case 2: restricted constant and no trend			
Dependent variable: *D(ngdp_g)*			
*D(bm_g)*	0.070670	0.882189	0.3819
*D(bm_g(-1))*	− 0.477649***	− 5.776245	0.0000
*ecm(-1)*	− 0.854291***	− 10.95162	0.0000

Diagnostic tests	Test statistic	Probability
Normality	0.162926	0.922
Heteroscedasticity (Breusch-Pagan-Godfrey)	F-statistic: 2.042	0.077
	Obs* R-squared 11.217	0.082
Breusch-Godfrey Serial correlation LM test (2 lags)	F-statistic: 0.5748	0.5666
	Obs*R-squared: 1.3332	0.5134
Ramsey RESET Test	F-statistic 0.211765	0.6474
CUSUM	stable	
CUSUM SQ	stable	

(*) and (***) denote the rejection of the null hypothesis at 10% and 1% levels of significance, respectively

**Table 6:** Long run and short run forms: specification 2

Variable	Coefficient	t-statistic	Prob. Value
Long run results: case 2: restricted constant and no trend			
Dependent variable: *ln_rgdp*			
*ln_gov_cons*	0.480224***	5.615976	0.0000
*ln_rbm*	0.247116***	4.506030	0.0001
*ln_rexport*	0.206142***	4.059953	0.0002
*constant*	4.939058***	4.958204	0.0000
EC = *ln_rgdp-(0.4802*ln_rgov_cons* + *0.2471ln_rbm* + *0.2061*ln_rexport* + *4.9391)*			
Short run results (ECM Form): case 2: restricted constant and no trend			
Dependent variable: *D(ln_rgdp)*			
*D(ln_rgov_cons)*	0.158940***	5.513950	0.0000
*D(ln_rgov_cons(*− *1))*	0.111161***	3.545886	0.0011
*D(ln_rgov_cons(*− *2))*	− 0.119017***	− 3.884408	0.0004
*D(ln_rbm)*	0.054726	1.981196	0.0550
*D(ln_rbm(*− *1))*	− 0.091657***	− 2.878767	0.0066
*D(ln_rbm(*− *2))*	0.035741	1.040842	0.3047
*D(ln_rbm(*− *3))*	0.075950**	2.217469	0.0328
*D(ln_rexport)*	0.049112***	3.686207	0.0007
*D(ln_rexport(*− *1))*	− 0.038099**	− 2.371036	0.0231
*D(ln_rexport(*− *2))*	− 0.050009***	− 2.999342	0.0048
*ecm(*− *1)*	− 0.187284***	− 5.846741	0.0000

Diagnostic tests	Test statistic	Probability
Normality	1.890654	0.388553
Heteroscedasticity (Breusch-Pagan-Godfrey)	F-statistic: 0.464389	0.9538
	Obs* R-squared: 9.671626	0.9168
Breusch-Godfrey Serial Correlation LM test (2 lags)	F-statistic: 1.186136	0.3174
	Obs*R-squared: 3.491225	0.1745
Ramsey RESET Test	F-statistic: 3.077343	0.0879
CUSUM	stable	
CUSUM SQ	stable	

(***) and (**) denote the rejection of the null hypothesis at 1% and 5% levels of significance, respectively

## Footnotes

1 It is worth mentioning, however, that some studies that adopt the structural models find that monetary policy is more effective than fiscal policy in terms of the impact on GDP (see for instance, Yunanto and Medyawati (2014) on the Indonesian economy).

2 www.cbe.gov.eg

3 The original St. Louis equation as appeared in Andersen and Jordan (1968) took the following form**:** ΔY_t_ = α + ${\sum }_{i=0}^{n}$ β_i_ΔM_t-i_ + ${\sum }_{i=0}^{n}$ δ_i_ΔE_t-i_ + ${\sum }_{i=0}^{n}$ ɣ_i_ΔR_t-i_ + ξ_t._ Where α is a constant; Y, M, E and R refer to nominal GNP, narrow money stock, high employment government expenditure, and high employment government receipts, respectively; the symbol Δ refers to the first difefrence; ξ refers to the error term assumed to be normally distributed with zero mean and constant variance, and n equals 3. It is noteworthy that the original study estimated other specifications of this equation. In one of the alternative specifications, the monetary base (B) is used instead of the money stock. In another specification, high employment budget surplus (R-E) is used as the fiscal measure instead of high employment expenditure and receipts.
